# Mr. Knowles's Case of Ascites

**Published:** 1810-08

**Authors:** Edward Lloyd Knowles

**Affiliations:** Newmarket


### *To the Editors of the Medical and Physical Journal.*

Gentlemen,

A Married Lady, about 30 years of age, of a plethoric habit, was affected in January 1803, with the usual symptoms of ascites; which, in the opinion of the two medical men who attended her, originated from a hard and long protracted labour, as it was perceived to augment gradually upon her from the above period; during the time the lymph was accumulating she again conceived, and went her regular time, and was delivered of a full-grown live fœtus. About two months after the birth of this child, and after the various medicaments had been administered, according to the discretion of the medical attendants, without the least perceptible benefit being derived, the tension of the abdomen becoming almost insupportable, paracentesis was performed; by which means, sixteen pints of a thick glutinous fluid were evacuated. What medicines were given immediately after, I was not able to learn; but whatever was administered, had not the least effect in exciting the action of the absorbents, or in preventing the effusion of lymph. She once more became pregnant, and after being in that state for three months, she was attacked with a most violent and excruciating pain of the body, extending across the left hypochondrium; she had a slight nausea at the time, with little or no thirst, and the pain extending upwards to the scapula. The medical gentlemen who first attended her were divided in opinions; one being certain that a disease of the liver existed, independant of the ascites; and the other as certainly asserted it to be a mis-placement of the same viscus. At this time, which was in March 1804, about fourteen months from the commencement of the affection, I was requested to visit her; the pulse was very languid, the system much emaciated, and she was in a forward state of pregnancy. After the cessation of the pain which affected the left hypochondrium, a violent soreness of the abdomen was the consequence, and, from this time, I dated the origin of an indurated tumour of some of the viscera; upon which I imagined the continuance of the malady mostly depended. The pain being situated immediately in the left hypochondrium, gave me a supposition it had no connection with the liver, but was a morbid enlargement of the spleen.

In

In consequence of the abdomen filling at the expence of every other secretion, and as the disease had been of long continuance, the system was so extremely debilitated, that I had but very little probability of success; and indeed, I considered it as an hopeless case, (but Providence deemed it otherwise). Notwithstanding this, I suggested a mode of procedure; the other gentlemen coinciding with me, as to its being appropriate to the case. As there was no time to lose, it was instantly executed, with an intention of removing the visceral obstructions.

Hab. Sub. mur. hydrarg. gr. ß. Pilul. sapon. gr. v. M. ft. bol. nocte maneque sumend. Hab. tinct. digital. purpur. gtt. xx. Super. tart. potass gr. xx. Decoct. cinchona, q. s. ft. haust. 8va. quaque hora sumend. Stimulating embrocations were applied to the abdomen, with friction, to augment the action of the decreased vessels, with an occasional intervention of the following:

℞. Sub. mur. hydrarg gr. v. Pulv. rad. convolv. jalap ℈j. M. ft. pulv. pro re nata sumend.

By this mode of treatment, the enlargement of the spleen gradually diminished, and became less painful; the visceral obstructions were soon removed; the digitalis excited the action of the kidnies beyond my expectation; and a great discharge of urine followed; in the course of two months, the fluid was totally evacuated, (which is rather singular, after the operation had been performed, as then it generally is more obstinate to remove); a nutritious regimen to support the vis vitæ was ordered. By this mode of procedure, and the exhibition of some tonic medicines, that valuable gift (health) was gradually restored, and she has since been the mother of two children.

I am, &c.

EDWARD LLOYD KNOWLES.

*Newmarket, June 22, 1810.*

